# Evaluation of a large-scale reproductive, maternal, newborn and child health and nutrition program in Bihar, India, through an equity lens

**DOI:** 10.7189/jogh.10.021011

**Published:** 2020-12

**Authors:** Victoria C Ward, Yingjie Weng, Jason Bentley, Suzan L Carmichael, Kala M Mehta, Wajeeha Mahmood, Kevin T Pepper, Safa Abdalla, Yamini Atmavilas, Tanmay Mahapatra, Sridhar Srikantiah, Evan Borkum, Anu Rangarajan, Swetha Sridharan, Dana Rotz, Debarshi Bhattacharya, Priya Nanda, Usha Kiran Tarigopula, Hemant Shah, Gary L Darmstadt, for the Ananya Study Group, Yamini Atmavilas, Debarshi Bhattacharya, Jason Bentley, Evan Borkum, Suzan Carmichael, Indrajit Chaudhuri, Andreea Creanga, Gary L. Darmstadt, Priyanka Dutt, Laili Irani, Tanmay Mahapatra, Kala M. Mehta, Radharani Mitra, Wolfgang A. Munar, Priya Nanda, Kevin T. Pepper, Hina Raheel, Anu Rangarajan, Niranjan Saggurti, Padmapriya Sastry, Hemant Shah, Sridhar Srikantiah, Usha Kiran Tarigopula, Victoria Ward, Yingjie Weng, Dilys Walker, Jess Wilhelm

**Affiliations:** 1 Department of Pediatrics, Stanford University School of Medicine, Stanford, California, USA; 2 Quantitative Sciences Unit, Department of Medicine, Stanford University School of Medicine, Stanford, California, USA; 3 Center for Population Health Sciences, Stanford University School of Medicine, Palo Alto, California, USA; 4 Department of Epidemiology and Biostatistics, University of California San Francisco, San Francisco, California, USA; 5 Bill and Melinda Gates Foundation, Delhi, India; 6 CARE India, Patna, India; 7 Mathematica, Princeton, New Jersey, USA

## Abstract

**Background:**

Despite increasing focus on health inequities in low- and middle income countries, significant disparities persist. We analysed impacts of a statewide maternal and child health program among the most compared to the least marginalised women in Bihar, India.

**Methods:**

Utilising survey-weighted logistic regression, we estimated programmatic impact using difference-in-difference estimators from Mathematica data collected at the beginning (2012, n = 10 174) and after two years of program implementation (2014, n = 9611). We also examined changes in disparities over time using eight rounds of Community-based Household Surveys (CHS) (2012-2017, n = 48 349) collected by CARE India.

**Results:**

At baseline for the Mathematica data, least marginalised women generally performed desired health-related behaviours more frequently than the most marginalised. After two years, most disparities persisted. Disparities increased for skilled birth attendant identification [+16.2% (most marginalised) vs +32.6% (least marginalized), *P* < 0.01) and skin-to-skin care (+14.8% vs +20.4%, *P* < 0.05), and decreased for immediate breastfeeding (+10.4 vs -4.9, *P* < 0.01). For the CHS data, odds ratios compared the most to the least marginalised women as referent. Results demonstrated that disparities were most significant for indicators reliant on access to care such as delivery in a facility (OR range: 0.15 to 0.48) or by a qualified doctor (OR range: 0.08 to 0.25), and seeking care for complications (OR range: 0.26 to 0.64).

**Conclusions:**

Disparities observed at baseline generally persisted throughout program implementation. The most significant disparities were observed amongst behaviours dependent upon access to care. Changes in disparities largely were due to improvements for the least marginalised women without improvements for the most marginalised. Equity-based assessments of programmatic impacts, including those of universal health approaches, must be undertaken to monitor disparities and to ensure equitable and sustainable benefits for all.

**Study registration:**

ClinicalTrials.gov number NCT02726230

With the transition from the Millennium Development Goals to the Sustainable Development Goals (SDGs), equity and the mandate to “leave no one behind” has become central to the global health agenda [1]. A call to “reduce inequality within and among countries” was explicitly articulated in the SDGs alongside the ambitious target of universal coverage of health services [2]. Similarly, the Addis Ababa Declaration of 2012 called for the promotion of equity in all health interventions globally, “to achieve the highest possible standards of health for all” [3]. Thus, in the SDG era, increasing attention is being paid to the monitoring of inequities and social determinants of health when evaluating the impact of global health interventions.

Health inequities have been found to be particularly large in reproductive, maternal, newborn and child health and nutrition (RMNCHN) services in low- and middle-income counties (LMICs) [4-6]. Research focused on evaluating inequities in population coverage of key evidence-based RMNCHN interventions in LMICs [7-9] demonstrates major differences in coverage and impact across population sub-groups; coverage of key health services remains low for the most marginalised groups [5]. Intersecting factors such as gender, early age of marriage, caste, religion and socioeconomic status have all been shown to moderate the utilisation of health services and the impact of interventions [10]. When evaluating the differential impacts of programs, multiple studies have shown that while important advances have been made in equity for exclusive breastfeeding and immunisation coverage [11,12], significant gaps remain, especially for interventions that rely on access to facility-based care such as skilled birth attendance and antenatal care (ANC) visits [13].

Despite the importance of reducing inequities in RMNCHN services in LMICs, especially for reductions in maternal and child mortality [14], few studies have rigorously evaluated changes in inequity from multi-dimensional interventions at scale. To address this evidence gap, we undertook an equity analysis of a statewide, six-year RMNCHN program (2012-2017) in Bihar, India.

## METHODS

### Study setting and context

In 2009, India accounted for 20% of the world’s population and a disproportionately high percentage of global maternal deaths [15,16]. India also had high measures of income inequality, as reflected by the GINI Index [17]. Bihar is among India’s most populous states with an estimated 104 million people and some of the highest rates of poverty (39% below the poverty line) and illiteracy (about 63% among females) in the world [18]. In 2006, the National Family Health Survey (NFHS-3) showed that rates of key RMNCHN-related health behaviours were also exceedingly low including ≥4 ANC visits (11%), institutional deliveries (20%), exclusive breastfeeding (28%) and contraceptive prevalence rate (34%) (Table S1 in the **Online Supplementary Document**) [18].

Against this backdrop, the Bill and Melinda Gates Foundation (BMGF) funded the development and implementation of a RMNCHN program called *Ananya*. The program promoted innovations in interventions and delivery platforms with the goal of supporting the government to improve the quality, uptake and equity of key health behaviours amongst women in Bihar, as described previously [19]. Innovations in intervention delivery designed by non-governmental organisation (NGO) partners were piloted in governmental health systems in eight districts, representing approximately one-quarter of Bihar’s population (28 million) with a plan to support the Government of Bihar (GoB) to scale up successful solutions across all 38 districts and 104 million people.

### Objective

We undertook an equity analysis to test the hypothesis that the health impacts of the program would not differ for those women who were most marginalised compared to those who were least marginalised, given the program’s intent to achieve universal coverage. We analysed two distinct sources of data, and utilised an intersectionality approach described by Sen et al [20] in order to understand the ways in which multiple causes of marginalisation influence health impacts. To discern whether the program led to increased or decreased equity in health-related behaviours [19], we set out to examine whether: 1) there were disparities in indicators at baseline; and 2) the disparities changed over the course of the program during the piloting and scale-up phases.

### Data sources and study population

Data sources for this study included Mathematica evaluation data and Community-based Household Surveys (CHS).

#### Mathematica data

**Figure 1:**
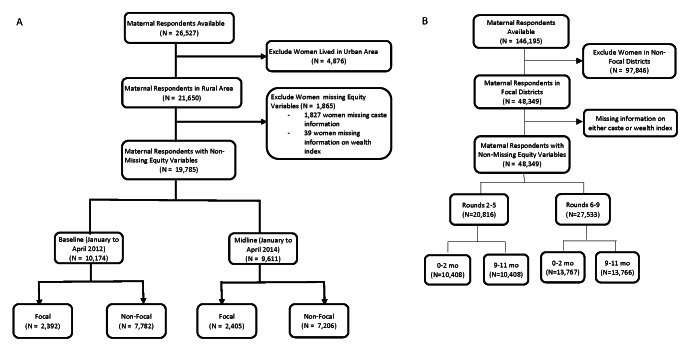
Study flow diagrams. **Panel A.** Mathematica surveys, Bihar, India, 2012 and 2014. **Panel B.** CHS surveys, Bihar, India, 2012-2017.

Mathematica implemented a statewide household evaluation at two time points: January through April 2012 (“baseline”) and January through April 2014 (“midline”), as described previously [21]. Survey data were collected from maternal household respondents in the eight focus districts of implementation as well as the 30 districts where *Ananya* was not implemented as a comparison. In both survey rounds, Mathematica conducted a listing to identify women who had given birth in the previous 12 months (about 13 women per village, on average). Thus, the 2012 (n = 10 174) and 2014 (n = 9611) surveys represented repeated cross-sections of mothers. Surveys focused on children ages 0-11 months because health interventions were targeted most intensively on pregnancy and on infant health in the first year after delivery. We excluded women who lived in urban areas (18%), given the significant differences between rural and urban wealth indices including ownership of household assets, household construction materials and sources of drinking water. **Figure 1**, Panel A provides a study flow diagram for the Mathematica data.

#### CHS data

The CHS data were collected across nine rounds of a household survey for community-based program monitoring, as described previously [22]. The surveys were designed to be a monitoring tool for CARE India and GoB staff to provide district-level point estimates, as well as block-level pass/fail results for a range of indicators at the household level. A design sampling methodology similar to “Lot Quality Assurance Sampling” was used in rounds 2 to 5 (phase I) and a modified version, “Lot Quality Assurance Sampling plus,” was applied in rounds 6 to 9 (phase II) [23]. We excluded round one, as it was a pilot survey to establish the methodology. Rounds 2 to 9 of the surveys were carried out between 2012 and the end of 2017. Phase I of the surveys (rounds 2 to 5) overlapped with the Mathematica evaluation period and with the eight-district pilot phase of implementation. Program scale up began between rounds five and six, and Phase II surveys (rounds 6 to 9) were conducted during the period when governmental implementation was extended statewide to all 38 districts with technomanagerial support of the Bihar Technical Support Program [19]) We included maternal respondents in the eight focus districts where data were available across all rounds (2 to 9) of the surveys. **Figure 1**, Panel B provides a study flow diagram for the CHS data.

### Study outcomes

Indicators, selected prior to analysis, were pertinent to interventions across the continuum of care, relevant to health disparities, and comparable with other analyses reported previously [21,22]. For each continuum of care domain – including antenatal, delivery, postnatal, family planning, immunisation and nutrition – we further classified the indicators by delivery platform. This included frontline worker (FLW) performance or behaviour, mother’s behaviour, and facility care and outreach service delivery, as described previously [19,21,22]. Our aim was to characterise program impact based on continuum of care domains and delivery platforms in order to examine trends for subgroups of indicators relevant to program implementation (**Table 1**). Indicators were chosen prior to analysis by three independent members of the Stanford analytic team with expertise in maternal and child health and the conduct of field trials. This process yielded 19 indicators for analysis, 18 and 15 of which were available in the CHS data and the Mathematica data, respectively.

**Table 1:** Reproductive, maternal, newborn and child health and nutrition indicators by continuum of care domains (rows) and intervention delivery platforms (columns) for the Mathematica and Community-based Household Survey data

	FLW performance or behaviour	Mother’s behaviour	Facility outreach / service delivery
**Antenatal care**	• Received iron-folic acid	• Pregnancy registration • Sought care for complications	• 4+ antenatal care visits
**Birth preparedness**		• Arranged transportation to facility • Identified skilled birth attendant	
**Family planning**	• FLW advised sterilisation • FLW advised PPIUD post-delivery		
**Delivery**	• FLW advised handwashing by delivery attendant		• Qualified doctor conducted facility delivery • Facility (public or private) delivery
**Postnatal care**	• FLW visited day or next day after delivery/return from hospital • FLW advised skin-to skin care	• Skin-to-skin care	
**Complementary feeding/nutrition**	• FLW advised exclusive breastfeeding	• Initiation of complementary feeding	• Immediate breastfeeding
**Immunisation**			• DPT3 by card

DPT – diphtheria, pertussis, tetanus, FLW – frontline worker, PPIUD – postpartum intrauterine device

### Equity domains

Given the well-known impacts of socioeconomic status and class on health outcomes [24,25], we defined equity domains based on the intersectionality of wealth tertiles and three Indian caste categories: Scheduled Caste/Scheduled Tribe (SCST), Other Backward Caste, and General/Other [20]. Caste was recorded during both CHS and Mathematica surveys (Figure S1 in the **Online Supplementary Document**). We created wealth indices from information collected by Mathematica and CHS for household assets, house construction materials, source of drinking water and education using principal component analysis (PCA), patterned after the National Family Health Survey (NFHS)-4 wealth index for rural areas [26]. For CHS rounds 2 to 5, a shorter list of items was available, similar to those used in the NFHS-3 wealth index (Table S2 in the **Online Supplementary Document**) [18,27]. This approach allowed us to maximise the number of items used to generate a wealth index while being as consistent as possible across the two data sources and with existing NFHS wealth indices. Table S3 in the **Online Supplementary Document** lists the items used in PCA by data source, and compares the loading factors for each item for each index (Mathematica, CHS rounds 2 to 5, and CHS rounds 6 to 9) compared with NFHS-3 and NFHS-4 rural. For analysis, we divided the wealth scores into tertiles. We then created a 9-level variable from the intersection of the three caste categories and the wealth tertiles. We refer to the two, clearly identified extreme groups – SCST/Wealth Tertile 1 and General-Other/Wealth Tertile 3 – as the most and least marginalised, respectively; comparison of these groups was the focus of our analysis.

### Analysis

We first described the demographic characteristics of women by data source, overall and among the least and most marginalised women.

#### Mathematica data

Using Mathematica data, we set out to assess whether there were disparities in health-related behaviours between the least and most marginalised groups at baseline. We then examined differences in program impact on RMNCHN health-related behaviours between the two groups by estimating separate survey logistic regressions for each group. Each survey-weighted logistic regression included an interaction term between the eight focus (intervention) districts (compared to the 30 non-focus districts as referent) and survey time (with baseline as referent), yielding a difference-in-difference (DID) estimator of program impact which corresponds to the interaction term. Models were adjusted for women’s age and gender of the focal child. The survey-weighted logistic regressions also included stratum and sampling weights at the village level (primary sampling unit) and used finite population corrections. As all covariates used in adjustment were included as categorical variables, any missing values were included as a level in the variable. We report the DID as an absolute percentage point change for each group and provide its *P*-value. In addition, we assessed whether the estimated DIDs were different between the most and least marginalised groups by fitting a combined model for both groups and adding a three-way interaction term between treatment group (focus or comparison districts), survey time (baseline or midline) and equity group (most or least marginalised). We applied a Benjamini-Hochberg False Discovery Rate (FDR) correction to this set of *P* values to account for multiple testing [28].

#### CHS data

In order to assess the equity impacts over time, we examined the CHS data set to compare the indicators between the least and most marginalised groups for each survey round (2 to 9). To do so, for each indicator we estimated a separate survey logistic regression for each round that included the intersection variable (ie, most vs least marginalised), mothers’ age, and gender of the focal child. The survey logistic regressions also included sample weights for each block (sampling stratum), where sample weights were the inverse of the number of women sampled divided by the eligible block population. Block populations were derived from Census rural block population estimates and crude birth rates [16,29]. We report both absolute rates with 95% confidence intervals (CIs) for indicators for the most and least marginalised as well as odds ratios (ORs) comparing the two groups. We also applied a Benjamini-Hochberg false discovery ate (FDR) correction to this set of *P*-values [28].

To assess whether changes in the ORs over time were different for the most vs least marginalised groups for each indicator, we examined an additional survey-weighted logistic regression model that included round (as an ordinal variable), the intersection variable, and an interaction term between round and the intersection variable, adjusted for mother’s age and gender of the focal child. Three sets of models were fit, one using data from all rounds, the second using pilot Phase 1 rounds 2 to 5, and the third using scale-up Phase 2 rounds 6 to 9, so that linear trends in the equity estimates could be evaluated for all rounds, as well as separately in phase I pilot phase and the phase II statewide scale-up period, as described previously [22]. A Benjamini-Hochberg correction was similarly applied to the *P* values for all ORs to account for multiple testing [28]. Statistical analyses were performed using SAS 9.4 and R 3.4.3 (IBM Inc, Armonk, NY, USA).

### Ethical considerations

Permission for access and terms of CHS data use were agreed upon with CARE India through a data sharing agreement. Analysis of CHS and Mathematica data was approved by the Stanford University Institutional Review Board protocol 39719. This study is part of the *Ananya* program which was registered with ClinicalTrials.gov number NCT02726230.

## RESULTS

### Study population demographics

The final cohort for analysis included 19 785 women from two Mathematica surveys (baseline, midline) and 48 349 women from eight rounds of CHS (**Figure 1**). Women respondents on average had similar proportions of illiteracy, no schooling, >2 children in the household, and male focal children in the Mathematica and the CHS cohorts (**Table 2**). There were lower proportions of large households (46.5% vs 69.3%) and non-nuclear families (44.9% vs 61.6%) in the Mathematica compared to the CHS data.

**Table 2:** Demographic characteristics of maternal respondents in the Mathematica and Community-based Household Surveys (CHS) by least and most marginalised groups in Bihar, India

	Mathematica – baseline (2012) and midline (2014)			CHS (rounds 2 to 9, 2012-2017)		
	**All**	Least Marginalised*	**Most Marginalised†**	**All**	Least Marginalised*	**Most Marginalised †**
**No.**	**19785**	**1279 (6.5%)**	**2681 (13.6%)**	**48349**	**3082 (6.4%)**	**6110 (12.6%)**
**Age, years:**						
<21	2394 (12.1)	142 (11.1)	308 (11.5)	8477 (17.5)	438 (14.2)	1027 (16.8)
21-25	9385 (47.4)	698 (54.6)	1125 (42.0)	23 023 (47.6)	1685 (54.7)	2610 (42.7)
26-30	6014 (30.4)	368 (28.8)	896 (33.4)	12 627 (26.1)	750 (24.3)	1756 (28.7)
31-35	1413 (7.1)	52 (4.1)	239 (8.9)	3292 (6.8)	170 (5.5)	536 (8.8)
>35	578 (2.9)	19 (1.5)	113 (4.2)	930 (1.9)	39 (1.3)	181 (3)
**Literacy:**						
Illiterate	11784 (59.6)	218 (17.0)	2309 (86.1)	29 340 (60.7)	609 (19.8)	5436 (89)
Literate	8001 (40.4)	1061 (83.0)	372 (13.9)	19 009 (39.3)	2473 (80.2)	674 (11)
**Hindu**	18044 (91.2)	1253 (98.0)	2671 (99.6)	42 212 (87.3)	2333 (75.7)	6011 (98.4)
**Women’s education status:**						
No schooling	11492 (58.1)	203 (15.9)	2274 (84.8)	29 457 (60.9)	620 (20.1)	5447 (89.1)
Some schooling	8293 (41.9)	1076 (84.1)	407 (15.2)	18 889 (39.1)	2461 (79.9)	662 (10.8)
**Had a BPL card**	11763 (59.5)	514 (40.2)	1779 (66.4)			
**Nuclear family:**						
No	8885 (44.9)	775 (60.6)	777 (29.0)	29 791 (61.6)	2430 (78.8)	2674 (43.8)
Yes	9222 (46.6)	360 (28.1)	1691 (63.1)	18 558 (38.4)	652 (21.2)	3436 (56.2)
Missing	1678 (8.5)	144 (11.3)	213 (7.9)	0 (0)	0 (0)	0 (0)
**Small household (household size ≤5):**						
No	9195 (46.5)	628 (49.1)	1087 (40.5)	33 495 (69.3)	2305 (74.8)	3683 (60.3)
Yes	8912 (45.0)	507 (39.6)	1381 (51.5)	14 854 (30.7)	777 (25.2)	2427 (39.7)
Missing	1678 (8.5)	144 (11.3)	213 (7.9)	0 (0)	0 (0)	0 (0)
**Number of children in the household at end:**						
1	6106 (30.9)	559 (43.7)	677 (25.3)	13 262 (27.4)	1158 (37.6)	1318 (21.6)
2	5458 (27.6)	404 (31.6)	663 (24.7)	12 965 (26.8)	998 (32.4)	1431 (23.4)
3	3955 (20.0)	196 (15.3)	560 (20.9)	9944 (20.6)	518 (16.8)	1339 (21.9)
4+	4266 (21.6)	120 (9.4)	781 (29.1)	12 178 (25.2)	408 (13.2)	2022 (33.1)
**Gender of the focal child:**						
Female	9346 (47.2)	561 (43.9)	1311 (48.9)	23 095 (47.8)	1438 (46.7)	3046 (49.9)
Male	10 439 (52.8)	718 (56.1)	1370 (51.1)	25 254 (52.2)	1644 (53.3)	3064 (50.1)

BPL – below poverty line

*Least marginalised, defined as General/Other/highest wealth tertile.

†Most margnisalised defined as Scheduled caste/Scheduled trive (SCST)/lowest wealth tertile.

### Equity group distributions and characteristics

**Figure 2:**
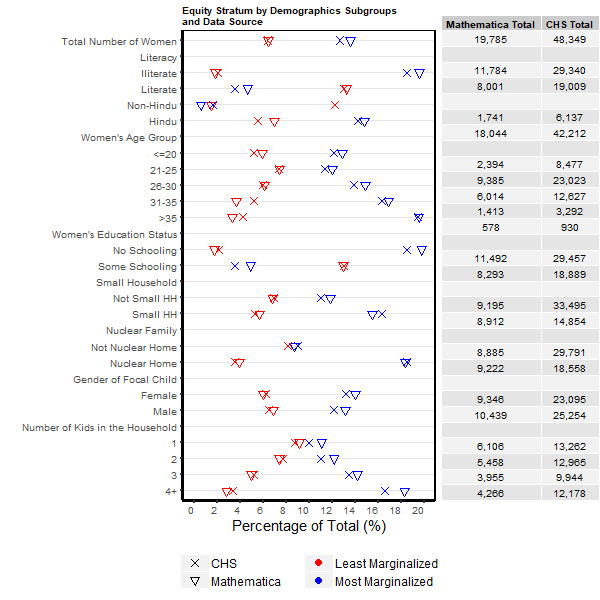
Comparisons of maternal respondents’ demographics by least vs most marginalised group for Mathematica (2012, 2014) and Community-based Household Surveys (CHS) data (2012-2017), Bihar, India.

The overall proportions of the study cohorts that were in the most marginalised group (13.6% and 12.6%) and the least marginalised group (6.5% and 6.4%) were similar for the Mathematica and CHS data, respectively (**Table 2**). For Mathematica data, the proportion in the most marginalised group was similar at baseline (13.3%) and midline (13.8%), while the least marginalised group increased slightly from baseline (5.7%) to midline (7.3%). Across CHS rounds, the most marginalised group varied between 8.8% to 16.5%, while the proportion of the cohort that was least marginalised generally remained the same (6%-7% across rounds). Examination of proportions of women classified as belonging to the most or least marginalised group for strata under each demographic variable showed consistent patterns across data sources (**Figure 2**). For women ages 21 and above, there was a general trend for increasing rates of women belonging to the most marginalised group with increasing age. Belonging to the most marginalised group was strongly associated with illiteracy and lack of education. A nuclear family, small household and an increasing number of children were all associated with increased likelihood of being in the most marginalised group. A female focal child was more likely to have a mother in the most marginalised group. Absolute rates of those in the most and least marginalised groups by characteristics were consistent between Mathematica and CHS data, with typically less than ± 5% relative deviation.

### Program impacts on equity by continuum of care domain

#### Mathematica data

Among the 15 indicators assessed using the baseline Mathematica data, we found that the least marginalised women generally performed health-related behaviours at a higher frequency across most indicators than the most marginalised, demonstrating that significant disparity existed at baseline favouring the least marginalised (**Table 3**). This was true across antenatal care, birth preparedness, delivery, immunisation and nutrition domains; the only exceptions were indicators for pregnancy registration and initiation of complementary feeding, which were similar for least and most marginalised women. The two groups showed similar frequencies across postnatal care indicators. Programmatic impact was significantly different for the most vs the least marginalised groups for some indicators after adjustment for multiple testing (ie, the difference in DIDs had *P* < 0.05), but for most indicators the disparity remained generally the same. For two indicators (**Table 3**), a lower increase was seen in focal districts from baseline to midline for the most marginalised compared to the least marginalised group, reflecting an increase in disparity during the program period. These indicators were: identification of a skilled birth attendant [+16.2% (most marginalised) vs +32.6% (least marginalized), *P* < 0.01) and skin-to-skin care (+14.8% vs +20.4%, *P* < 0.05). Of note, there was no significant change in disparity for advice given by a FLW regarding skin-to-skin care. For delivery in a facility, the most marginalised demonstrated a -1% decrease during the program period while the least marginalised demonstrated a +10.9% increase; however, these results were not statistically significant (*P* = 0.083). A closing of the equity gap was found for two indicators (**Table 3**) wherein the most marginalised in the focus districts demonstrated a significantly greater increase than the least marginalised: receipt of three doses of diptheria-pertussis-tetanus vaccine (DPT3) (-4.9% vs +3.5% for the least vs the most marginalised, respectively, *P* < 0.01%) and immediate breastfeeding (-4.9% vs +10.4% for the least vs the most marginalised, respectively, *P* < 0.01). For the former, the equity gap significantly closed, while for the latter, the equity gap was reversed.

**Table 3:** Comparison of *Ananya* pilot and scale-up program impact between least and most marginalised women based on Mathematica data, 2012 and 2014, Bihar, India

Indicator domain and indicator description	Equity group	Baseline			Midline								Difference-in-Difference (DID) estimate				*P*-value comparing DID estimates for least and most marginalised groups*,†		Result on equity	
**Focal**	**Non-focal**				**Focal**		**Non-focal**										
**Antenatal care (ANC)**																				
4+ antenatal care (ANC) visits	Least marginalised	51%		47%				49%		51%				-6.7%				0.690		
	Most marginalised	35%		34%				33%		26%				5.4%						
**Identified skilled birth attendant**	**Least marginalised**	**75%**		**68%**				**100%**		**60%**				**32.6%**				**<0.01**	**Widening of equity gap**	
	**Most marginalised**	**50%**		**48%**				**65%**		**47%**				**16.2%**						
Arranged transportation	Least marginalised	50%	46%			20%			16%			-0.1%						0.369			
	Most marginalised	3%	8%			7%			6%			6.7%									
Pregnancy registration	Least marginalised	71%	66%			73%			69%			-2.5%						0.711			
	Most marginalised	80%	83%			87%			93%			-2.5%									
Received ≥90 iron-folic acid tablets	Least marginalised	24%	28%			16%			20%						-0.4%			0.200			
	Most marginalised	13%	13%			15%			18%						-3.6%						
**Delivery**																					
Place of delivery: In a facility (public or private)	Least marginalised	81%	84%			93%			85%						10.9%			0.083			
	Most marginalised	53%	47%			62%			58%						-1.0%						
Qualified doctor conducted facility delivery	Least marginalised	38%	36%			37%			28%						6.7%			0.369			
	Most marginalised	11%	11%			12%			8%						4.8%						
**Immunisation**																					
**DPT3‡ recorded on immunisation card**	**Least marginalised**	**38%**	**43%**			**45%**			**55%**						**-4.9%**			**<0.01**	**Closing of equity gap**		
	**Most marginalised**	**29%**	**34%**			**39%**			**40%**						**3.5%**						
**Nutrition**																					
Frontline worker advised exclusive breastfeeding	Least marginalised	50%	49%			33%			30%						1.1%			0.629			
	Most marginalised	39%	32%			42%			27%						8.7%						
**Immediate breastfeeding**	**Least marginalised**	**51%**	**43%**			**52%**			**48%**						**-4.9%**			**<0.01**	**Reversal of equity gap**		
	**Most marginalised**	**43%**	**43%**			**57%**			**47%**						**10.4%**						
Initiation of complementary feeding	Least marginalised	65%	64%			68%			61%						5.6%			0.239			
	Most marginalised	68%	61%			71%			60%						4.8%						
**Postnatal care**																					
FLW advised skin-to-skin care	Least marginalised	36%	46%			37%			26%						21.2%			0.064			
	Most marginalised	33%	28%			38%			22%						10.2%						
FLW visited day or next of delivery / Return from hospital	Least marginalised	21%	16%			5%			3%						-2.8%			0.369			
	Most marginalised	19%	15%			10%			5%						0.9%						
FLW visited within 1 week after delivery	Least marginalised	27%	19%			10%			4%						-2.4%			0.083			
	Most marginalised	23%	19%			13%			7%						1.7%						
**Skin-to-skin care**	**Least marginalised**	**18%**	**24%**			**46%**			**32%**						**20.4%**			**0.018**	**Widening of equity gap**		
	**Most marginalised**	**17%**	**14%**			**42%**			**24%**						**14.8%**						

*By false discovery rate (FDR) adjustment for family-wise error of the *P* values in difference-in-difference analysis.

†Adjusted for women’s age and gender of focal child.

‡Diphtheria-pertussis-tetanus (DPT3).

#### CHS data

**Figure 3:**
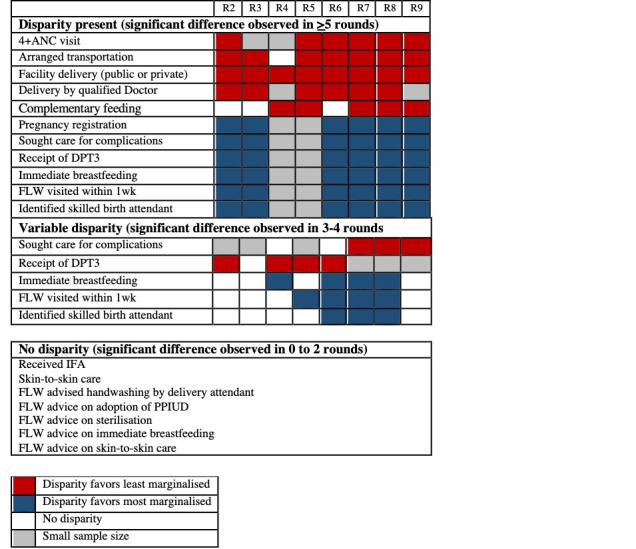
Comparison of the disparity across Community-based Household Surveys (CHS) indicators favouring the most marginalised vs the least marginalised, by round, Bihar, India, 2012-2017. ANC – antenatal care, DPT – diphtheria-pertussis-tetanus (DPT3), FLW – frontline worker, IFA – iron-folic acid, PPIUD – postpartum intrauterine device.

Among the 18 indicators analysed for CHS comparing the most marginalised to the least marginalised (as referent), most demonstrated disparity in frequency of behaviours between the two groups across rounds (**Figure 3****,** **Table 4**). Specifically, the OR between the most and least marginalised groups was significant for six indicators, primarily in antenatal care, birth preparedness and delivery-related indicators. During program implementation, indicators (n = 7) that showed consistently lower odds across rounds for the most marginalised group included 4+ ANC visits (OR range: 0.08 to 0.20), arranged transportation to facility (OR range: 0.40 to 0.64), sought care for complications (OR range: 0.26 to 0.64), delivery in a facility (OR range: 0.15 to 0.48), delivery by a qualified doctor (OR range: 0.08 to 0.25), receipt of DPT3 (OR range: 0.41 to 0.9), and initiation of complementary feeding (OR range: 0.34 to 0.94). Four indicators showed consistently higher odds for the most marginalised group: pregnancy registration (OR range: 1.68 to 6.3), identification of a skilled birth attendant (OR range: 1.25 to 2.72), immediate breastfeeding (OR range: 1.05 to 1.82) and FLW visited the day of or the day after delivery (OR range: 1.54 to 1.99).

**Table 4:** Comparison of Community-based Household Survey (CHS) indicators between the least and most marginalised by round 2-9, Bihar, India, 2012-2017*,†

Indicator	Round 2	Round 3	Round 4	Round 5	Round 6	Round 7	Round 8	Round 9
**Antenatal care**								
4+ antenatal care visits	**0.11 (0.05-0.25)**	N/A‡	N/A	**0.14 (0.08-0.27)**	**0.08 (0.05-0.15)**	**0.14 (0.09-0.21)**	**0.1(0.06-0.17)**	**0.2 (0.13-0.31)**
Identified skilled birth attendant	1.43 (0.81-2.53)	1.58 (0.98-2.56)	1.4 (0.86-2.25)	1.57 (0.98-2.52)	**1.79 (1.17-2.74)**	**2.13 (1.39-3.27)**	**2.72 (1.76-4.20)**	1.25 (0.77-2.04)
Arranged transport to facility	**0.47 (0.28-0.77)**	**0.41 (0.25-0.69)**	0.64 (0.34-0.88)	**0.54 (0.34-0.88)**	**0.57 (0.38-0.86)**	**0.4 (0.27-0.60)**	**0.56(0.37-0.85)**	**0.61(0.39-0.95)**
Sought care for complications	N/A	N/A	0.51 (0.24-1.11)	N/A	0.64 (0.30-1.40)	**0.33 (0.21-0.52)**	**0.26 (0.17-0.42)**	**0.43 (0.28-0.66)**
Pregnancy registration	**1.68 (1.02 -2.76)**	**2.12 (1.27-3.55)**	N/A	N/A	**2.89 (1.89-4.41)**	**6.3 (3.85-10.31)**	**3.76 (2.28-6.21)**	**3.72 (2.02-6.85)**
Received iron-folic acid tablets	1.03 (0.67-1.58)	0.92 (0.60-1.40)	1.12 (0.74-1.70)	1.46 (0.96-2.23)	**1.45 (1.00-2.10)**	**1.47 (1.05-2.09)**	0.8 (0.54-1.18)	1.1 (0.76-1.60)
Frontline worker (FLW) advised hand-washing by attendant	0.85 (0.49-1.47)	**1.78 (1.05-3.02)**	1.02 (0.60-1.76)	1.49 (0.85-2.62)	0.92 (0.55-1.52)	1.4 (0.81-2.42)	1.78 (0.98-3.25)	1.33 (0.78-2.27)
**Delivery**								
Delivery in a facility (public or private)	**0.22 (0.12-0.41)**	**0.48 (0.29-0.81)**	**0.17 (0.10-0.32)**	**0.24 (0.13-0.43)**	**0.27 (0.16-0.47)**	**0.15 (0.09-0.26)**	**0.18 (0.10-0.31)**	**0.21 (0.13-0.35)**
Facility delivery by Qualified Doctor	**0.25 (0.12-0.49)**	**0.22 (0.11-0.43)**	N/A	**0.08 (0.04-0.18)**	**0.12 (0.07-0.20)**	**0.16 (0.09-0.28)**	**0.10 (0.05-0.19)**	N/A
**Family planning**								
FLW advised adoption of PPIUD	0.85 (0.39-1.85)	0.93 (0.49-1.79)	**0.47 (0.24-0.90)**	1.25 (0.62-2.48)	**0.45 (0.25-0.81)**	1.46 (0.67-3.15)	1.81 (0.86-3.85)	0.57 (0.29-1.12)
FLW advised adoption of sterilisation	1.24 (0.67-2.33)	1.21 (0.66-2.22)	0.66 (0.36-1.22)	**2.52 (1.37-4.64)**	0.73 (0.42-1.27)	**2.21 (1.06-4.60)**	1.4 (0.73-2.70)	1.09 (0.59-2.03)
**Immunisation**								
DPT3 recorded on immunisation card	**0.41 (0.27-0.62)**	0.9 (0.63-1.35)	**0.51 (0.33-0.81)**	**0.63 (0.42-0.96)**	**0.48 (0.33-0.69)**	N/A	N/A	N/A
**Nutrition**								
Initiation of complementary feeding	0.92 (0.61-1.38)	0.94 (0.62-1.43)	**0.39 (0.21-0.75)**	**0.46 (0.22-0.94)**	0.91 (0.60-1.38)	**0.34 (0.19-0.59)**	**0.52 (0.35-0.75)**	**0.34 (0.20-0.55)**
Immediate breastfeeding	1.3 (0.86-1.98)	1.35 (0.90-2.03)	**1.82 (1.20-2.76)**	1.42 (0.94-2.17)	**1.8 (1.25-2.59)**	**1.74 (1.23-2.47)**	**1.75 (1.22-2.50)**	1.05 (0.68-1.64)
FLW advised exclusive breastfeeding	0.63 (0.35-1.13)	0.84 (0.48-1.47)	0.86 (0.49-1.50)	1.52 (0.84-2.75)	0.66 (0.39-1.11)	0.98 (0.57-1.66)	1.33 (0.72-2.44)	1.11 (0.66-1.87)
**Postnatal care**								
Skin-to-skin care	1.14 (0.64-2.05)	0.9 (0.53-1.54)	0.64 (0.37-1.10)	1.32 (0.81-2.15)	1.53 (0.96-2.45)	**2.16 (1.21-3.89)**	1.31 (0.82-2.08)	**1.71 (1.06-2.75)**
FLW advised skin-to-skin care	0.76 (0.42 -1.36)	1.13 (0.65-1.95)	1.09 (0.63-1.89)	1.35 (0.77- 2.38)	1.18 (0.68-2.08)	1.1 (0.57-2.12)	1.27 (0.68-2.41)	1.08 (0.62-1.90)
FLW visited within 1 week after delivery	0.9 (0.59-1.36)	1.38 (0.92-2.08)	1.14 (0.76-1.71)	**2.99 (1.95-4.59)**	**1.69 (1.15-2.49)**	**2.06 (1.43-2.98)**	**2.52 (1.69-3.74)**	1.28 (0.89-1.84)

DPT – diphtheria, pertussis, tetanus; PPIUD – postpartum intrauterine device

*Adjusted for women’s age and gender of focal child.

†Expressed as an odds ratio comparing the most to the least marginalised women as referent, with 95% confidence interval.

‡Number of women in the subgroups of either the least or most marginalised group for the indicator was less than 10 and thus was not reported in this table.

No significant linear trends of the ORs over rounds were found for any of the indicators. Generally there were no significant changes in linear trends for the most marginalised across indicators assessed and thus, any changes in disparity were driven by changes in trends for the least marginalised (Figure S2 in the **Online Supplementary Document**). For three indicators, improvement in the behaviours of the least marginalised led to increased disparity compared to the most marginalised, including delivery by a qualified doctor, initiation of complementary feeding, and receipt of DPT3 (**Table 5**). For four indicators, a decrease in the behaviour by the least marginalised group led to increased disparity favouring the most marginalised, including pregnancy registration, identification of a skilled birth attendant, visit by a FLW within one week of delivery, and immediate breastfeeding. Disparity persisted but trends did not change over time for 4+ ANC visits, arrangement of transportation to a facility, facility delivery, and seeking care for complications. There were no significant disparities observed for indicators related to FLW-delivered advice, as well as skin to skin care and receipt of iron-folic acid (IFA).

**Table 5:** Categorisation of trends over time for least marginalized* compared to the most marginalised† based on Community-based Household Survey (CHS) data, Bihar, India (2012-2017)

**Increased disparity due to least marginalised improvement:**	**Increased disparity due to least marginalised worsening:**
• Delivery by a qualified doctor • Initiation of complementary feeding • Receipt of diptheria, pertussis, tetanus (DPT)3	• Pregnancy registration • Identified skilled birth attendant • Immediate breastfeeding • Frontline worker (FLW) visited within 1 week of delivery
**Disparity stable over time, favoring least marginalized:**	**No disparity, no significant changes over time:**
• 4+ antenatal care visits • Arranged transport to facility • Sought care for complications • Facility delivery	• Received iron-folic-acid tablets • Skin-to-skin care • FLW advised handwashing • FLW advised post-partum intrauterine device • FLW advised sterilisation • FLW advised breastfeeding • FLW advised skin to skin care

*Least marginalised (LM) defined as General/Other/highest wealth tertile.

†Most marginalised (MM) defined as SCST/lowest wealth tertile.

## DISCUSSION

The *Ananya* program aimed to improve the health of women across Bihar through governmental delivery of RMNCHN interventions by means of FLW outreach, improved health messaging, and improved facility-based care. At baseline, women’s behaviours often did not adhere to recommendations for continuum of care services which have been proven effective for reductions in maternal and neonatal morbidity. The program aimed to improve this, with varying success [21,22,30,31]. In this analysis, we aimed to assess whether there were disparities in impact for these RMNCHN indicators throughout the course of implementation, for those women who were most marginalised compared to the least marginalised.

With regard to the two groups assessed, we found that generally the most marginalised women were older, less literate or educated, had more children and a nuclear family. A female focal child was also more likely to have a mother in the most marginalised group.

The results of our equity analysis demonstrated that there were disparities in many health-related behaviours between the most and least marginalised mothers at baseline in 2012, and for the most part these disparities persisted throughout the study period. CHS data suggested that the largest disparities favouring the least marginalised were observed for those indicators dependent on access to care, such as delivery in a facility, arrangement of transportation to a facility, and seeking care for complications. For some indicators, improvements were seen for the least marginalised over time, which were not seen for the most marginalised, thus leading to increases in disparities. These indicators included delivery by a qualified doctor, initiation of complementary feeding and receipt of DPT3, and thus could also be considered as related to access to care or to varied food sources. In contrast, indicators that demonstrated improvements in equity over time typically were behaviours that were driven primarily by maternal choice such as registration of pregnancy, identification of a skilled birth attendant, and immediate breastfeeding. Given that there were no significant disparities observed for indicators related to FLW advice between the two groups, the CHS data suggest that inequities were more likely due to access to care rather than to differences in exposure to health messaging.

These findings align with prior studies suggesting that interventions delivered through health facilities, such as skilled birth attendance and ANC visits are significantly more prone to inequity than community-based interventions or those initiated by individual agency such as breastfeeding [5,13]. The drivers of these findings are likely multifactorial, and include individual-level issues of access (including geographic, social and financial barriers to access such as time poverty and household support), system-level issues of inadequate health financing, limited human resources, and social determinants of health such as discrimination driven by caste and economic disadvantage [6].

The Mathematica data suggests a more mixed picture, although notably the midline data was collected only two years after program implementation began. Similar to the CHS data, the most marginalised group demonstrated greater improvement in immediate breastfeeding. In contrast to CHS data, greater improvements were seen in the least marginalised women in maternal behaviours of skin-to-skin care and identification of a skilled birth attendant. Those who were most marginalised had increased improvement in DPT3 rates. These improvements of the most marginalised are consistent with prior studies which report the least inequities in impact for breastfeeding and immunisation rates [4,5,11].

There are several limitations to this study. All data sets relied on women’s self-reported behaviours and thus are at risk for response and social desirability biases. Second, women were surveyed from different geographical areas, and thus may have had varying exposures to program interventions and there may have been cluster effects amongst communities of women. Furthermore, while the Mathematica survey was the most rigorous of the evaluations conducted as surveys were administered by evaluators who were independent of implementation and the data are further strengthened by its DID framework, data collection occurred after a relatively short, two-year period of program implementation. The CHS surveys, on the other hand, were collected over six years of implementation but were intended to be used for internal monitoring information rather than for program evaluation. Therefore, they were collected without a comparison group and thus could not account for secular improvements in the indicators occurring concurrently. Further, the round 2 survey was collected after implementation of some interventions had already begun and thus did not serve as a true baseline.

The most notable limitation in our equity analysis was its narrowed scope of comparison to the least and most marginalised groups, or the extreme ends of the equity spectrum rather than across the intermediary groups, due to the challenges inherent in an intersectionality approach. As a term, “intersectionality” focuses on the ways in which interactions of multiple social determinants and inequalities lead to health inequities [20,32-34]. However, there is no agreement in the literature on how to prioritise across contributing sociodemographic and economic variables; rather, intersectionality “rejects hierarchical ordering of oppression” [35]. Therefore, the ordering of these intermediary groups would be impacted by the varying contributions of individual contextual factors over time and across geographic settings, and would also differ across indicators [36,37]. For that reason, this analysis focused on those subcategories which were defensibly at the ends of the spectrum, enabling us to elucidate more reliably how socioeconomic inequalities influence the impact of the program across indicators, time and geography.

Despite these limitations, the results suggest that the *Ananya* program had variable impact on the inequity that existed at baseline between the least and most marginalised groups across the continuum of care and delivery platforms. While disparities were observed across most health-related behaviours – and these disparities generally persisted over time – there were significant increases in inequity between these two groups, most notably among those indicators reliant upon access to care. Examination of reasons for this in the context of Bihar is warranted as part of a comprehensive effort to reduce health inequities, which could include increased investment in improving contributing factors such as affordability, accessibility and quality of care, including dignified treatment and gender equality [6,10,14]. From an equity perspective, evidence from the literature also suggests that strengthening community-based care may be beneficial. A review by Schleiff et al. analysed the equity effects of programs utilising community-based primary health care and found that community-based projects had greater success in decreasing inequity for maternal and child health due to their accessibility when compared to projects that strengthened services at facilities [13].

By providing support across FLW outreach, community and facility-based platforms, the *Ananya* program intended to improve health for all Bihari women similar to that of a universal health approach. We show that the program’s impacts were largely seen in the improvement or worsening of indicators among the least marginalised, with relatively little changes amongst the most marginalised. These findings suggest that some health interventions, like those delivered through health facilities, may have been inaccessible to, or could not be acted upon by those who were most disadvantaged. On the contrary, those facets of the *Ananya* program in which the most marginalised women were specifically identified and targeted, such as the *Parivartan* self-help group program, demonstrated successful results [30,31]. In Parivartan, self-help groups were leveraged to empower hard-to-reach and marginalised women, which led to significant improvements across RMNCHN indicators.

Together, this body of data demonstrates the importance of recent calls for universal health programs to assess equity impacts [5,6,14], as programs that take an equity blind approach in their implementation may be at risk for perpetuating or even exacerbating inequities. Instead, underlying disparities must be measured and addressed, even targeted, with progress monitored throughout implementation. Additional research may be required, including mixed methods approaches, to understand the contributing factors and underpinning reasons for inequities, and to monitor programmatic impact on the most marginalised groups as well as across intermediary groups. Only then can large-scale health interventions ensure that they are closing gaps of disparity, and thus upholding the SDGs’ call to “leave no one behind.”

## CONCLUSION

While there is an ever-increasing armamentarium of evidence-based RMNCHN interventions, these interventions require further evaluation through an equity lens. Advances will not achieve sustainable benefits for future generations without ensuring that sub-populations that differ along intersecting axes of social determinants of health also benefit equitably. Further, these analyses must inform the adoption and implementation of programs and policies worldwide. This will not only require increased funding, but also political investment in equity. As noted by Alkebrack et al., greater political commitment through government spending is the biggest predictor of health equity [38]. Governments must be willing to invest in equity-oriented systems to promote inclusive health advancements.

As we advance technology, expand the evidence for cost-effective interventions, and scale-up RMNCHN in LMICs, it will be important to focus on rigorous study of disparities in health indicators between subgroups, and the factors underlying these disparities. This will ensure that investments in global health benefit all communities, particularly those who may need them the most.

## Additional material

Online Supplementary Document

## References

[1] WHO. From Millennium Development goals to Sustainable Development Goals: the situation and trends in 2015. Geneva: World Health Organization; 2015.

[2] United Nations. Transforming our world: the 2030 Agenda for Sustainable Development New York, NY: United Nations; 2015.

[3] Asnake M, Bishaw T. The Addis Ababa Declaration on Global Health Equity: A call to action. Ethiopian Journal of Health Development. 2012;233-37 p.

[4] Victora CG Barros AJ Axelson H Bhutta ZA Chopra M Franca GV How changes in coverage affect equity in maternal and child health interventions in 35 Countdown to 2015 countries: an analysis of national surveys. Lancet. 2012;380:1149-56. 10.1016/S0140-6736(12)61427-5 22999433

[5] Barros AJ Ronsmans C Axelson H Loaiza E Bertoldi AD Franca GV Equity in maternal, newborn, and child health interventions in Countdown to 2015: a retrospective review of survey data from 54 countries. Lancet. 2012;379:1225-33. 10.1016/S0140-6736(12)60113-5 22464386

[6] Boerma T Requejo J Victora CG Amouzou A George A Agyepong I Countdown to 2030: tracking progress towards universal coverage for reproductive, maternal, newborn, and child health. Lancet. 2018;391:1538-48. 10.1016/S0140-6736(18)30104-1 29395268

[7] Darmstadt GL Bhutta ZA Cousens S Adam T Walker N de Bernis L Evidence-based, cost-effective interventions: how many newborn babies can we save? Lancet. 2005;365:977-88. 10.1016/S0140-6736(05)71088-6 15767001

[8] Bhutta ZA Das JK Bahl R Lawn JE Salam RA Paul VK Can available interventions end preventable deaths in mothers, newborn babies, and stillbirths, and at what cost? Lancet. 2014;384:347-70. 10.1016/S0140-6736(14)60792-3 24853604

[9] Lassi ZS, Kumar R, Bhutta ZA. Community-Based Care to Improve Maternal, Newborn, and Child Health. In: Black RE, Laxminarayan R, Temmerman M, Walker N, editors. Reproductive, Maternal, Newborn, and Child Health: Disease Control Priorities, Third Edition (Volume 2). Washington DC: The International Bank for Reconstruction and Development / The World Bank; 2016. 27227219

[10] McDougal L Atmavilas Y Hay K Silverman JG Tarigopula UK Raj A Making the continuum of care work for mothers and infants: Does gender equity matter? Findings from a quasi-experimental study in Bihar, India. PLoS One. 2017;12:e0171002. 10.1371/journal.pone.0171002 28146586 PMC5287473

[11] Roberts T Carnahan E Gakidou E Can breastfeeding promote child health equity? A comprehensive analysis of breastfeeding patterns across the developing world and what we can learn from them. BMC Med. 2013;11:254. 10.1186/1741-7015-11-254 24305597 PMC3896843

[12] Brearley L Eggers R Steinglass R Vandelaer J Applying an equity lens in the Decade of Vaccines. Vaccine. 2013;31 Suppl 2:B103-7. 10.1016/j.vaccine.2012.11.088 23598470

[13] Schleiff M Kumapley R Freeman PA Gupta S Rassekh BM Perry HB Comprehensive review of the evidence regarding the effectiveness of community-based primary health care in improving maternal, neonatal and child health: 5. equity effects for neonates and children. J Glob Health. 2017;7:010905. 10.7189/jogh.07.010905 28685043 PMC5491949

[14] Carrera C Azrack A Begkoyian G Pfaffmann J Ribaira E O’Connell T The comparative cost-effectiveness of an equity-focused approach to child survival, health, and nutrition: a modelling approach. Lancet. 2012;380:1341-51. 10.1016/S0140-6736(12)61378-6 22999434

[15] Kassebaum NJ Bertozzi-Villa A Coggeshall MS Shackelford KA Steiner C Heuton KR Global, regional, and national levels and causes of maternal mortality during 1990-2013: a systematic analysis for the Global Burden of Disease Study 2013. Lancet. 2014;384:980-1004. 10.1016/S0140-6736(14)60696-6 24797575 PMC4255481

[16] Special SRS. Bulletin on Maternal Mortality 2007-2009. New Delhi: Registrar General of India, Ministry of Home Affairs, Government of India; 2011.

[17] World Bank. GINI index. Data. Washington DC: World Bank; 2017.

[18] National Family Health Survey (NFHS-3). 2005–06. International Institute for Population Sciences; India, Mumbai. 2007.

[19] Darmstadt GL Pepper KT Ward VC Srikantiah S Mahapatra T Tarigopula UK Improving primary health care delivery in Bihar, India: Learning from piloting and statewide scale-up of *Ananya.* J Glob Health. 2020;10:021001 . 10.7189/jogh.10.021001 PMC7757841 33414906

[20] Sen G Iyer A Mukherjee C A Methodology to Analyse the Intersections of Social Inequalities in Health. J Human Dev Capabil. 2009;10:397-415. 10.1080/19452820903048894

[21] Darmstadt GL Weng Y Pepper KT Ward V Mehta KM Borkum E Impact of the *Ananya* program on reproductive, maternal, newborn and child health and nutrition in Bihar, India: early results from a quasi-experimental study. J Glob Health. 2020;10:021002 10.7189/jogh.10.021002 PMC7757842 33427822

[22] Abdalla S Weng Y Mehta KM Srikantiah S Mahapatra T Shah H Trends in reproductive, maternal, newborn and child health and nutrition indicators during five years of piloting and scaling-up of *Ananya* interventions in Bihar, India. J Glob Health. 2020;10:021003 10.7189/jogh.10.021003 PMC7757843 33427818

[23] Robertson SE Valadez JJ Global review of health care surveys using lot quality assurance sampling (LQAS), 1984-2004. Soc Sci Med. 2006;63:1648-60. 10.1016/j.socscimed.2006.04.011 16764978

[24] Balarajan Y Selvaraj S Subramanian SV Health care and equity in India. Lancet. 2011;377:505-15. 10.1016/S0140-6736(10)61894-6 21227492 PMC3093249

[25] Bora JK Raushan R Lutz W The persistent influence of caste on under-five mortality: Factors that explain the caste-based gap in high focus Indian states. PLoS One. 2019;14:e0211086. 10.1371/journal.pone.0211086 31430275 PMC6701792

[26] National family health Survey (NFHS-4), 2015-16. Mumbai: International Institute for Population Sciences; 2017.

[27] Bassani DG Corsi DJ Gaffey MF Barros AJ Local distributions of wealth to describe health inequalities in India: a new approach for analyzing nationally representative household survey data, 1992-2008. PLoS One. 2014;9:e110694. 10.1371/journal.pone.0110694 25356667 PMC4214688

[28] Hochberg Y Benjamini Y More powerful procedures for multiple significance testing. Stat Med. 1990;9:811-8. 10.1002/sim.4780090710 2218183

[29] Annual Health Survey 2010-2011. Ministry of Home Affairs; 2011.

[30] Mehta KM Irani L Chaudhuri I Mahapatra T Schooley J Srikantiah S Health impact of self-help groups scaled up statewide in Bihar, India. J Glob Health. 2020;10:021006 10.7189/jogh.10.021006 PMC7761401 33425330

[31] Ward VC Raheel H Weng Y Mehta KM Dutt P Mitra R Impact of mHealth interventions for reproductive, maternal, newborn and child health and nutrition at scale: BBC Media Action and the *Ananya* rogram in Bihar, India. J Glob Health. 2020;10:021005 10.7189/jogh.10.021005 PMC7758913 33425329

[32] Iyer A Sen G Ostlin P The intersections of gender and class in health status and health care. Glob Public Health. 2008;3 Suppl 1:13-24. 10.1080/17441690801892174 19288340

[33] Dey A Hay K Afroz B Chandurkar D Singh K Dehingia N Understanding intersections of social determinants of maternal healthcare utilization in Uttar Pradesh, India. PLoS One. 2018;13:e0204810. 10.1371/journal.pone.0204810 30286134 PMC6171889

[34] Bowleg L The problem with the phrase women and minorities: intersectionality-an important theoretical framework for public health. Am J Public Health. 2012;102:1267-73. 10.2105/AJPH.2012.300750 22594719 PMC3477987

[35] Hankivsky O Women’s health, men’s health, and gender and health: implications of intersectionality. Soc Sci Med. 2012;74:1712-20. 10.1016/j.socscimed.2011.11.029 22361090

[36] Bauer GR Incorporating intersectionality theory into population health research methodology: challenges and the potential to advance health equity. Soc Sci Med. 2014;110:10-7. 10.1016/j.socscimed.2014.03.022 24704889

[37] Sridharan S Dey A Seth A Chandurkar D Singh K Hay K Towards an understanding of the multilevel factors associated with maternal health care utilization in Uttar Pradesh, India. Glob Health Action. 2017;10:1287493. 10.1080/16549716.2017.1287493 28681668 PMC5533144

[38] Alkenbrack S Chaitkin M Zeng W Couture T Sharma S Did Equity of Reproductive and Maternal Health Service Coverage Increase during the MDG Era? An Analysis of Trends and Determinants across 74 Low- and Middle-Income Countries. PLoS One. 2015;10:e0134905. 10.1371/journal.pone.0134905 26331846 PMC4558013

